# METTL3 mediates chemoresistance by enhancing AML homing and engraftment via ITGA4

**DOI:** 10.1038/s41375-022-01696-w

**Published:** 2022-10-20

**Authors:** Mingying Li, Jingjing Ye, Yuan Xia, Meng Li, Guosheng Li, Xiang Hu, Xiuhua Su, Dongmei Wang, Xin Zhao, Fei Lu, Jingxin Li, Daoxin Ma, Tao Sun, Chunyan Ji

**Affiliations:** 1 Department of Hematology, Qilu Hospital of Shandong University, Cheeloo College of Medicine, Shandong University, Jinan, 250012 People’s Republic of China; 2 Shandong Key Laboratory of Immunohematology, Qilu Hospital of Shandong University, Cheeloo College of Medicine, Shandong University, Jinan, 250012 People’s Republic of China; 3 School of Medicine, Cheeloo College of Medicine, Shandong University, Jinan, 250012 China; 4 Department of Physiology, School of Basic Medical Sciences, Cheeloo College of Medicine, Shandong University, Jinan, 250012 People’s Republic of China

**Keywords:** Leukaemia, Cell signalling, Cancer microenvironment

## Abstract

Chemoresistant leukemia relapse is one of the most common causes of death for acute myeloid leukemia (AML) patients and the homing/engraftment in bone marrow (BM) are crucial steps for AML cells to acquire chemoresistance by interacting with stromal cell components. No crosstalk between m^6^A modification and homing/engraftment has been reported. Here, we performed comprehensive high-throughput analyses, including RNA sequencing of CR (complete remission) and relapsed AML patients, and reverse-phase protein arrays of chemoresistant cells to identify METTL3 as a key player regulating AML chemoresistance. Then, METTL3-mediated m^6^A modification was proved to induce the chemoresistance in vitro and in vivo. Furthermore, AML homing/engraftment was discovered being enhanced by upregulated-METTL3 in chemoresistant cells. And the homing/engraftment and drug-resistance associated phenotypes of chemoresistant cells could be reversed by a METTL3 inhibitor. Mechanistically, METTL3 extended the half-life of ITGA4 mRNA by m^6^A methylation, and then, increased expression of ITGA4 protein to enhance homing/engraftment of AML cells. The results provide insights into the function of m^6^A modification on the interaction between AML cells and BM niches and clarify the relationship between METTL3 and AML homing/engraftment, suggesting a therapeutic strategy for the treatment of refractory/relapsed AML with METTL3 inhibitors.

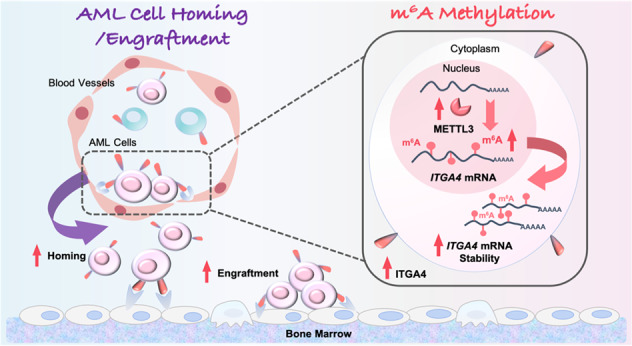

## Introduction

Acute myeloid leukemia (AML) is a hematologic malignancy characterized by dysregulated proliferation and accumulation of leukemic cells in the hematopoietic system [1]. Induction therapy for AML is dependent on intensive chemotherapy. However, many patients experience poor outcomes following traditional chemotherapies [2]. Many factors have been reported to be involved in the development of AML chemoresistance: ABC transporters [3], apoptotic dysregulation [4], gene mutations [5] and the bone marrow (BM) niche [6]. Studies on these factors have attempted to interpret the occurrence of chemoresistance from different aspects, but the precise mechanisms involved remain to be determined.

In mammalian cells, approximately 0.4% of all adenosines in mRNA are modified by m^6^A modification, which is the most prevalent internal chemical modification found in mRNAs [7]. m^6^A modification plays a key role in many physiological and pathological processes, such as viral infections, immune response, tissue regeneration, development of cancer cells and specification of hematopoietic or cancer stem cells [8–11]. Notably, m^6^A modification has been shown to be involved in the pathogenesis and development of AML by regulating the different fates of various mRNAs [12–16]. Although m^6^A methylation had been proved associating with cell adhesion [17, 18], no report about the relationship between m^6^A methylation and the homing/engraftment of AML cells in BM has been found.

METTL3 is the core methyltransferase of the m^6^A modification system [19]. It has been identified as an oncogene for AML [12, 20] and plays a role in regulating hematopoietic stem cell (HSC) differentiation [21]. Many efforts have been made to target METTL3 in the treatment of AML [22], but the relationship between METTL3 and AML chemoresistance has not been reported. In this study, we found that the expression of METTL3 in AML cells was related to the treatment outcome and proved that METTL3 mediated chemoresistance of cells and homograft and xenograft mouse models of AML. Moreover, METTL3 enhanced the AML homing/engraftment in an m^6^A-dependent manner. Then, a METTL3 inhibitor, STM2457, was proven to significantly reverse the homing/engraftment and drug-resistance of chemoresistant cells. Mechanistically, METTL3 promoted the development of AML chemoresistance via the METTL3-m^6^A-ITGA4-homing/engraftment axis. Together, the data demonstrate an important role of METTL3 in AML chemoresistance, showing potential application prospects for METTL3-mediated m^6^A modification in the treatment of refractory/relapsed AML.

## Materials and methods

Cell lines, lentivirus infection, RPPA, Mouse studies and animal housing, EdU and CFA assays, cell migration and adhesion experiments, RT-PCR, m^6^A dot blot, LC-MS/MS for determination of the m^6^A/A ratio, RNA-seq and m^6^A-seq, mRNA stability, Western blot and Dual-luciferase reporter assays are described in the Supplementary Materials and Methods section.

### Clinical samples

BMMNCs were isolated from primary and relapsed AML patients. All the related procedures for collection of the samples of patients with AML were performed with the approval from the Research and Ethics Committee of Qilu Hospital of Shandong University. All patients who provided clinical specimens signed the written informed consent form. All the procedures were performed in accordance with the 1964 Declaration of Helsinki principles and its later amendments or comparable ethical standards. CD34+ cells were isolated by CD34 MicroBeads according to the manufacturer’s instructions (Miltenyi Biotec, Germany, #130-046-702).

### MeRIP qPCR

The MeRIP Kit (Bersin Bio, China, #Bes5203) was used following the manufacturer’s instructions. Briefly, 200 mg of total RNA was sheared to approximately 300 nt in length by metal ion-induced fragmentation and purified with 450 µL of IP buffer with RNase inhibitors. One-ninth of the fragmented RNA was saved as an input control and then incubated with an m^6^A antibody (Synaptic Systems, #202003) or rabbit IgG with Protein A/G Magnetic Beads at 4 °C for 1 h. After washing with IP buffer, the m^6^A IP portion was eluted with 200 μL of elution buffer with Proteinase K at 55 °C for 45 min and purified. qPCR was performed along with the MeRIP-ed RNAs.

## Results

### METTL3 may play a role in AML chemoresistance

**Fig. 1:**
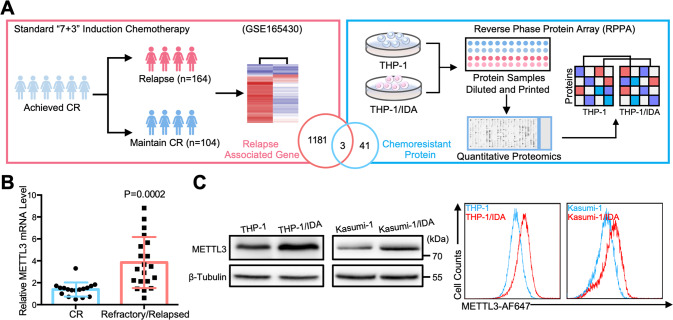
Up-regulation of METTL3 in chemoresistant AML cells. **A** Flow chart for the identification and verification strategy of differentially expressed targets between chemotherapy-resistant AML cells and -sensitive AML cells. Graphical elements were adapted from the RNA-seq (GSE165430, *n* = 268, Log_2_ FC ≥ 2) and RPPA screen (LogFC > 0.25) workflow. Significant: *P* < 0.05; nonsignificant: *P* ≥ 0.05. See also Supplemental Fig. S1. **B** qRT-PCR analysis of METTL3 mRNA expression in CD34 + leukemia cells sorted from pretreatment patients and relapsed patients with de novo AML. Pretreatment patients who achieved CR after induction treatment with standard “7 + 3” chemotherapy were defined as the CR group (*n* = 17), and NR patients and relapsed patients were defined as the refractory/relapsed group (*n* = 20). Data are mean ± SD values. *P* < 0.05 was considered significant, *t* test. **C** Western blot analysis and Flow cytometry analysis for the expression of METTL3, *n* = 3.

To identified targets related to chemotherapy resistance with potential for clinical application, we firstly analyzed RNA sequencing (RNA-seq) data from the GSE165430 dataset (Supplementary Table 1), which includes pretreatment samples from 268 adults with de novo cytogenetically normal AML (CN-AML) who were younger than 60 years of age and achieved complete remission (CR) after induction treatment with the standard “7 + 3” chemotherapy [23]. To narrow down the list of target genes related to AML chemoresistance, we then used reversed-phase protein array (RPPA) technology to obtain potential targets at the protein expression levels for clinical application. A total of 308 protein and phosphorylated protein targets involved in many cancer- and drug target-related cell signaling pathways were analyzed, and 44 proteins were significant raised in the IDA-resistant THP-1 cells (THP-1/IDA cells) were identified (Fig. 1A and Supplementary Fig. S1C). PDGFRB, METTL3 and IGFBP2 were identified as candidates related to AML chemoresistance at the mRNA and protein expression levels (Fig. 1A). Notably, a variety of FLT3 inhibitors whose potential inhibitory targets contain PDGFRB have entered clinical trials in refractory/relapsed AML [24]. Furthermore, the high expression of IGFBP2 is associated with AML chemoresistance and is an independent factor for the prediction of relapse of AML [25, 26]. Therefore, METTL3 would be with further research value to be a potential clinical treatment target for refractory/relapsed AML.

Then, we examined the relationship between METTL3 expression and AML chemoresistance, and found that the expression of METTL3 in most relapse AML patients was increased compared with that at primary diagnosis (GSE83533) (Supplementary Table 2). Furthermore, our clinical samples from pretreatment patients and relapsed patients with de novo AML showed that METTL3 was expressed at a significantly higher level in the refractory/relapsed group than in the CR group (Fig. 1B). The results confirmed that the expression of METTL3 was related to the poor prognosis of AML patients. Then, we generated drug-resistant AML cells, namely THP-1&THP-1/IDA cells and Kasumi-1&Kasumi-1/IDA cells, and we proved their dose response to chemotherapy (Supplementary Fig. S1D). We next studied the expression of METTL3 in the chemoresistant AML cell lines (THP-1&THP-1/IDA, Kasumi-1&Kasumi-1/IDA and HL-60&HL-60/ADR cells). The results showed that the METTL3 mRNA (Supplementary Fig. S1E, G) and protein (Fig. 1C, Supplementary Fig. S1F, H) levels in the three kinds of chemoresistant AML cell lines were actually overexpressed. Collectively, the data demonstrated that higher METTL3 expression correlates with AML chemoresistance.

Moreover, we enhanced the endogenous expression of METTL3 in THP-1 and Kasumi-1 cells by using the dCas9-SAM platform (Supplementary Fig. S2A). As expected, METTL3 overexpression by METTL3 gRNAs caused more AML cells to enter the cell division cycle (Supplementary Fig. S2B, C), which was consistent with the findings in a previous report [27] and suggested that METTL3 enhanced the proliferation of AML cells without chemotherapy treatment. More importantly, in the presence of IDA, a stronger ability of METTL3 to promote the proliferation of AML cells was observed (Supplementary Fig. S2B, C), suggesting that METTL3 conferred chemoresistance to AML cells. Moreover, in the METTL3-overexpressing group, the rate of decline in the number of colonies between the PBS and IDA subgroups decreased significantly compared with that of the cells in the NC group (Supplementary Fig. S2D, E); these results were consistent with the EdU incorporation assay results. These results confirmed that METTL3 promoted the proliferation of AML cells and that this effect was more obvious after treatment with chemotherapeutic drugs.

### METTL3 induces AML chemoresistance in an m^6^A catalytic activity-dependent manner

**Fig. 2:**
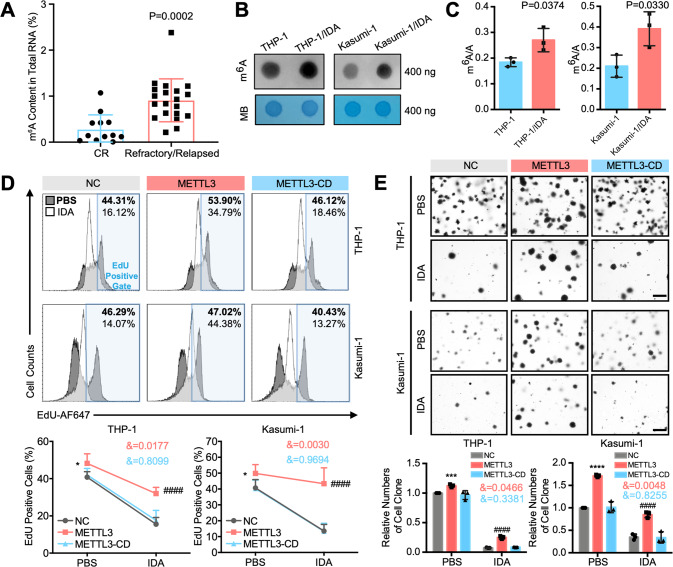
METTL3-meditated m^6^A enhances the chemoresistance of AML cells. **A** AML CD34+ cells from the patients shown in Fig. 1B were subjected to an m^6^A methylation quantification kit to assess global m^6^A changes (*n* = 13 for CR group and *n* = 20 for Refractory/Relapsed group). *P* < 0.05 was considered significant, *t* test. **B**, **C** m^6^A dot blot assays (**B**) and LC-MS/MS (**C**) for the detection of global m^6^A changes. MB, methylene blue staining (as a loading control). *P* < 0.05 was considered significant, *t* test. **D** EdU incorporation assay (upper) showing the percentage of AML cells that entered the proliferation cycle (EdU positive cells) with or without IDA pressure. Percentages after PBS treatment are shown in bold, and percentages after IDA treatment are shown in regular. Statistical analysis is shown in (lower). *P < 0.05, vs. the NC group with PBS treatment; ^####^*P* < 0.0001, vs. the NC group with IDA treatment; &, significant interaction effect; two-way ANOVA. **E** Colony-forming assays (upper). Bar, 500 μm. Statistical analysis is shown in (lower). ***P < 0.001, ****P < 0.0001, vs. the NC group with PBS treatment; ^####^*P* < 0.0001, vs. the NC group with IDA treatment; &, significant interaction effect; two-way ANOVA. *n* ≥ 3, mean ± SD values are shown for (**A**) and (**C**–**E**).

To determine whether the stimulatory effect of METTL3 on AML chemoresistance was caused by m^6^A modification, we analyzed the m^6^A modification levels in our clinical AML samples, as shown in Fig. 1B. The m^6^A levels in the patients in the refractory/relapsed group were significantly higher than those in the patients who achieved CR after two cycles of induction treatment (Fig. 2A). Moreover, m^6^A dot blot assays (Fig. 2B and Supplementary Fig. S1I) and liquid chromatography-tandem mass spectrometry (LC-MS/MS) (Fig. 2C) confirmed an increased levels of m^6^A in the mRNA from THP-1/IDA and Kasumi-1/IDA cells. The results suggested that METTL3 may regulate AML chemoresistance by improving m^6^A modification of mRNA.

Subsequently, a catalytic mutant of METTL3 (METTL3-CD, D394A and W397A, Supplementary Fig. S3A) was constructed [28]. METTL3 and METTL3-CD lentiviruses were packaged and transfected into THP-1 and Kasumi-1 cells. Then, the overexpression of METTL3 or METTL3-CD (Supplementary Fig. S3B) and their ability to promote m^6^A modification (Supplementary Fig. S3C) were verified. Furthermore, our results showed that METTL3-CD also lost its superiority in promoting cellular growth (Fig. 2D) and clonogenic ability (Fig. 2E) of human leukemia cells caused by METTL3 in the presence or absence of IDA. In addition, METTL3 significantly reduced the apoptosis of AML cells after IDA treatment, while METTL3-CD lost this function (Supplementary Fig. S3D). These results proved that METTL3 the enhancement of AML chemoresistance by METTL3 was depending on its m^6^A catalytic activity.

### METTL3-mediated m^6^A modification reduces the sensitivity of AML cells to chemotherapeutics agents in vivo

To determine whether METTL3 enhances AML chemoresistance in vivo, we established two mouse models of AML. In a xenotransplantation mouse model, THP-1 cells overexpressing METTL3 or METTL3-CD were intravenously injected into NOD-Prkdcscid-Il2rgem1IDMO (NSG) mice. Forty-two days later, IDA was injected intraperitoneally for 7 days to treat the disease (Supplementary Fig. S4A). In a homograft model, we isolated murine BM MLL-AF9+ AML cells from mice with established MLL-AF9 leukemia. After transfection with lentivirus carrying METTL3 or METTL3-CD, the MLL-AF9 leukemic cells were intravenously injected into C57BL/6 mice to establish secondary BM transplantation (BMT). Doxorubicin (DOX) and arabinosylcytosine (Ara-C) were injected beginning on the 8th day for 5 days (Supplementary Fig. S5A).

**Fig. 3:**
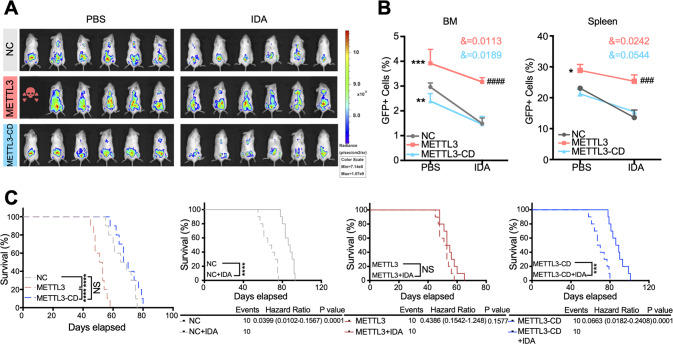
METTL3-meditated m^6^A reduces the sensitivity of AML cells to chemotherapeutics in vivo. **A** Representative in vivo pseudocolor bioluminescence images of NSG mice transplanted with control or METTL3/METTL3-CD THP-1 cells by detecting up the GFP signal. Unit of radiance is photons/second/cm^2^/steradian. **B** The distribution of THP-1 cells in BM and spleen of the NSG mice with or without IDA treatment was measured by flow cytometry at the end point. *P < 0.05, **P < 0.01, ***P < 0.001, vs. the NC group with PBS treatment; ^**###**^*P* < 0.001, ^####^*P* < 0.0001, vs. the NC group with IDA treatment; &, significant interaction effect; two-way ANOVA. **C** Kaplan–Meier survival curves (*n* = 10 for each group) showing the effects of forced METTL3 and METTL3-CD expression on the progression of human AML cells in NSG mice with or without IDA treatment. ***P < 0.001, ****P < 0.0001; NS nonsignificant. *n* ≥ 5, mean ± SD values are shown for (**B**).

The in vivo results showed that, after PBS treatment, the proportion of AML cells in BM and spleen in the METTL3 group was dramatically higher than that in the NC or METTL3-CD group. After IDA treatment, METTL3 also increased the proportion of AML cells, and the rate at which this proportion decreased in the IDA-treated group compared with the PBS-treated and METTL3-overexpressing group was significantly slower than that of the NC or METTL3-CD group (Fig. 3A, B; Supplementary Fig. S4C, D, Fig. S5, B–D). Furthermore, METTL3-overexpressing AML cells markedly increased the size of the spleen (Supplementary Figs. S4B and S5E) and exhibited greater infiltration into the live (Supplementary Figs. S4E and S5F) after IDA treatment than after PBS treatment, whereas the METTL3-CD group did not exhibit these phenomena. Finally, the survival of the animals was analyzed. In the METTL3-overexpressing groups, decreased survival was observed in the mice that were not treated with chemotherapy, demonstrating that the overexpression of METTL3 significantly promoted the progression of AML in recipients. Notably, no difference in lifespan was observed between the untreated and IDA-treated subgroups (Fig. 3C and Supplementary Fig. S5G), indicating that the therapeutic effect of IDA was blocked by the overexpression of METTL3 and strongly suggesting that METTL3 played a significant role in enhancing AML chemoresistance in the long term. To assess the pro-chemoresistance activity of METTL3 in a clinically relevant setting, we established patient-derived xenografts (PDXs), in which leukemic blast cells from the bone marrow of six AML patients were intravenously transplanted into NSG mice. We found that treatment of PDXs with IDA resulted in a slight decrease in CD45+ infiltration in the high METTL3 expression group and a significant decrease in the low METTL3 expression group (Supplementary Fig. S4F). The results from the AML xenograft and homograft models confirmed that METTL3 enhances chemoresistance by improving m^6^A methylation in vivo.

### METTL3 enhances AML cell migration/adhesion in vitro and homing/engraftment in vivo

**Fig. 4:**
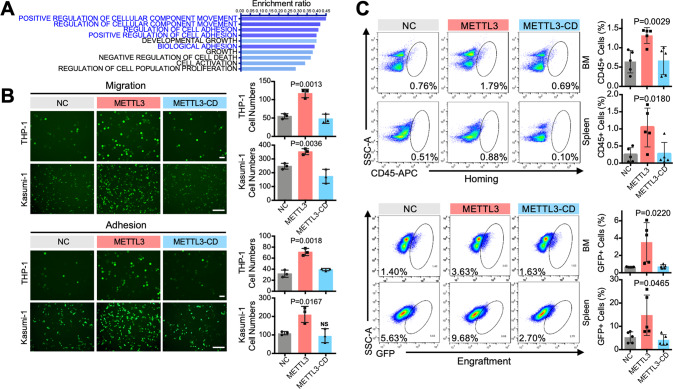
METTL3 enhances AML cell adhesion/migration in vitro and homing/engraftment in vivo. **A** GSEA analyses of changed genes from RNA-seq and m^6^A-seq analysis of METTL3-overexpressing and control THP-1 cells (*n* = 3). **B** Images (left) and corresponding statistical results (right) showing that METTL3-overexpressing AML cells significantly improved the migration (upper panel) and adhesion (lower panel) of AML cells. *P* < 0.05 was considered significant, vs. the NC group, *t* test. Bar, 250 μm. **C** Flow cytometry was carried out on the BM and spleen of the xenograft recipient mice 16 h (homing) or 42 days (engraftment) after tail vein injection. *P* < 0.05 was considered significant, vs. the NC group, *t* test. *n* ≥ 3, mean ± SD values are shown for (**B**) and (**C**).

To comprehensively understand the regulatory role of METTL3 in AML chemoresistance, we conducted RNA-seq and m^6^A sequencing (m^6^A-seq) in THP-1 cells. Our m^6^A-seq data showed that 3516 genes had significantly increased levels of m^6^A modification after METTL3 overexpression. Integrative analysis with the RNA-seq data identified 564 transcriptome-wide potential targets (Supplementary Fig. S6A) that were associated with significant changes in transcription with high confidence. Using the Molecular Signature Database (MSigDB) for gene set enrichment analysis (GSEA) [29], we identified 10 pathways in which genes with upregulated and downregulated expression were enriched (Fig. 4A) and found that METTL3 overexpression notably activated migration- and adhesion-related signaling pathways (Fig. 4A and Supplementary Fig. S6B). In addition, the METTL3 overexpression group had a higher m^6^A abundance on enriched gene transcripts involved in the migration/adhesion-related pathways (Supplementary Fig. S6C). Moreover, clustered growth was observed for METTL3-overexpressing AML cells (Supplementary Fig. S7A), and we hypothesized that METTL3 may enhance the migration and adhesion of AML cells. For verification, transwell migration assays and cell adhesion tests were carried out by coculturing with HUVECs. The results confirmed that METTL3 improved the migration and adhesion of AML cells, whereas METTL3-CD did not (Fig. 4B), which supported our hypothesis and indicated that METTL3 enhanced the migration and adhesion of AML cells by m^6^A modification.

In vivo, upon occupation of the hematopoietic niche and in a manner that is dependent on migration and adhesion, AML cells interact with BM stromal cells, maintain dormancy, increase their self-renewal activity, and develop drug resistance [30, 31]. Therefore, the homing and engraftment of METTL3-overexpressing AML cells in xenograft and homograft models were explored. For homing, the distribution of AML cells in the BM and spleen at 16 h after tail vein injection was studied (Supplementary Fig. S7B, F). Flow cytometry showed that METTL3 enhanced the homing of AML cells in the BM and spleen (Fig. 4C and Supplementary Fig. S7G), and immunohistochemistry assays of bone sections indicated that the proportion of METTL3-overexpressing AML cells close to the endosteum was significantly increased (Supplementary Fig. S7C). As expected, METTL3-CD had no effects on AML homing (Fig. 4C and Supplementary Fig. S7G). In addition, the engraftment results (42 days for xenotransplantation and 7 days for homotransplantation) showed that the expression of METTL3, but not METTL3-CD, increased the AML cell proportion in the BM and spleen (Fig. 4C, Supplementary Fig. S7D, G). Together, these results confirmed that METTL3 increased the homing and engraftment of AML cells in BM by m^6^A modification.

### A METTL3 inhibitor reverses AML chemoresistance and homing/engraftment capacity

**Fig. 5:**
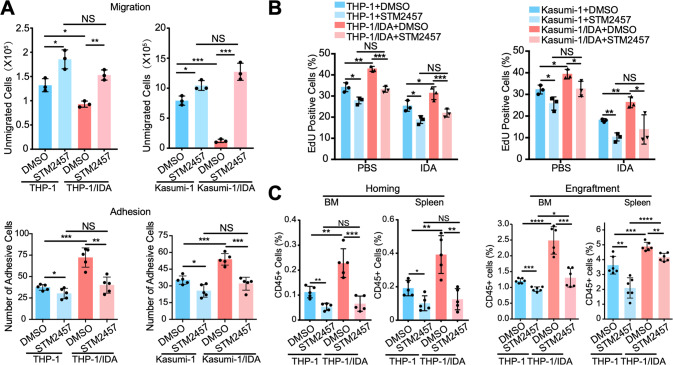
A METTL3 inhibitor reverses AML chemoresistance and homing/engraftment capacity. **A** Statistical results showing the improvement of migration (upper) and adhesion (lower) in THP-1/IDA and Kasumi-1/IDA cells can be reversed by STM2457 treatment of 48 h. *P < 0.05, **P < 0.01; ***P < 0.001, NS nonsignificant; *t* test. **B** The Statistical analysis of EdU incorporation assay showing the improvement of AML chemoresistance can be reversed by the pretreatment of STM2457. *P < 0.05, **P < 0.01; ***P < 0.001, NS nonsignificant. **C** The homing and engraftment of THP-1&THP-1/IDA cells with or without STM2457 treatment in the BM and spleen measured by flow cytometry 16 h and 42 days after tail vein injection. *P < 0.05, **P < 0.01; ***P < 0.001, ****P < 0.0001, NS nonsignificant; *t* test. *n* ≥ 3, mean ± SD values are shown for (**A**–**C**).

To explore the application prospect of STM2457 to treat AML chemoresistance, we treated chemoresistant AML cells with 10 μM STM2457 for 48 h and demonstrated its ability to reverse the increase in m^6^A modification (Supplementary Fig. S8A). In addition, we found that the migration and adhesion of chemoresistant AML cells were enhanced, but STM2457 inhibited these effects (Fig. 5A). Furthermore, STM2457-pretreatment significantly inhibited the proliferation of chemoresistant AML cells and promoted their apoptosis (Fig. 5B; Supplementary Fig. S8, B, C). Moreover, the corresponding inhibitory effect of STM2457 was observed, and the enhanced homing and engraftment (Fig. 5C and Supplementary Fig. S8, D–F) of chemoresistant AML cells were also reversed by STM2457-pretreatment before tail vein injection. Finally, we found STM2457-pretreated THP-1/IDA xenograft mice exhibited less AML cell infiltration in the BM and spleen after IDA treatment than controls (Supplementary Fig. S8G), suggesting that the inhibition of AML homing and engraftment caused by STM2457 pretreatment may rescue the sensitivity to IDA. These results confirmed that STM2457 could reverse the adhesion and homing of chemoresistant AML cells, thereby reducing the chemoresistance of AML cells. The METTL3 inhibitor would be a potential agent for chemoresistant AML patients.

### ITGA4 is the direct target of METTL3 in AML chemoresistance

**Fig. 6:**
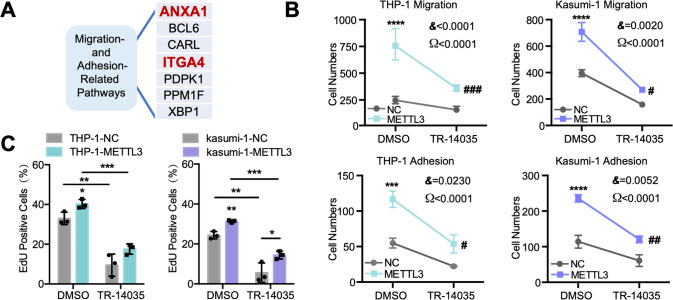
METTL3 mediates AML chemoresistance by regulating ITGA4. **A** ITGA4 appears at a high frequency in multiple migration and adhesion pathways. **B** The improvement of migration and adhesion in METTL3-overexpressing AML cells can be partly reversed by an ITGA4 inhibitor (TR-14035). ***P < 0.001, ****P < 0.0001, vs. the NC group under DMSO treatment; ^#^*P* < 0.05, ^##^*P* < 0^.^01, ^###^*P* < 0.001, vs. the NC group under TR-14035 treatment; Ω, significant TR-14035 treatment effect; &, significant interaction effect; two-way ANOVA. Bar, 200 μm. **C** EdU incorporation assay showing that the improvement in the percentage of METTL3-overexpressing AML cells entering the proliferation cycle with IDA pressure can be partly reversed by TR-14035. *P < 0.05, **P < 0.01; ***P < 0.001; *t* test. *n* ≥ 3, mean ± SD values are shown for (**B**) and (**C**).

To explore the key targets and elucidate the mechanisms underlying METTL3-mediated AML chemoresistance via m^6^A modification, we deeply analyzed and summarized the potential target genes in all migration- and adhesion-related pathways (Supplementary Table 3), identifying 7 potential targets that were common between these datasets. We further narrowed the intersection to 2 targets, ANXA1 and ITGA4, by considering the targets involved in the proliferation-related pathways that were among the 10 enriched pathways (Fig. 6A). ANXA1 is a macrophage-specific gene that related to inflammation-related migration [32], while ITGA4 is a classical molecule involved in AML homing [33, 34]. As an integrin, ITGA4 has been proven to be a central molecule by which AML cells binding to bone marrow stromal elements and that mediates cellular migration; ITGA4 may mediate anti-apoptotic signals and confer chemoresistance [35]. Thus, we decided to focus on ITGA4 for further studies. Then, the relationship between the expression of METTL3 and ITGA4 mRNA was analyzed using data from the TCGA database and our clinical samples. A positive correlation was observed (Supplementary Fig. S9A, B), suggesting that METTL3 is involved in promoting the expression of ITGA4. Therefore, the mRNA or protein expression of ITGA4 and its membrane surface distribution were further investigated (Supplementary Fig. S9C–F), and the results of ITGA4 expression was significantly increased when METTL3 was overexpressed in AML cells, which supported our hypothesis. Then, we found that ITGA4 expression in chemoresistant AML cells was increased compared with that in normal AML cells and that this change could be reversed by STM2457 (Supplementary Fig. S9G). Overall, these data indicated that METTL3 enhanced the expression of ITGA4. Then, we proved that the effects of METTL3 on promoting the migration and adhesion (Fig. 6B and Supplementary Fig. S9I) and chemoresistance of AML cells (Fig. 6C and Supplementary Fig. S9H) could be reduced by an ITGA4 inhibitor (TR-14035) [36, 37]. Notably, TR-14035 also reduced chemoresistance in the NC group, indicating that ITGA4 inhibitors further sensitized the cells to chemotherapy. Furthermore, we found that ITGA4 overexpression led to AML chemoresistance, which was similarly observed in the METTL3-CD group (Supplementary Fig. S9J). All the above results revealed the METTL3-m^6^A-ITGA4-homing/engraftment axis that contributes to the protection of bone marrow niches for AML cells

### METTL3 increases ITGA4 mRNA stability

**Fig. 7:**
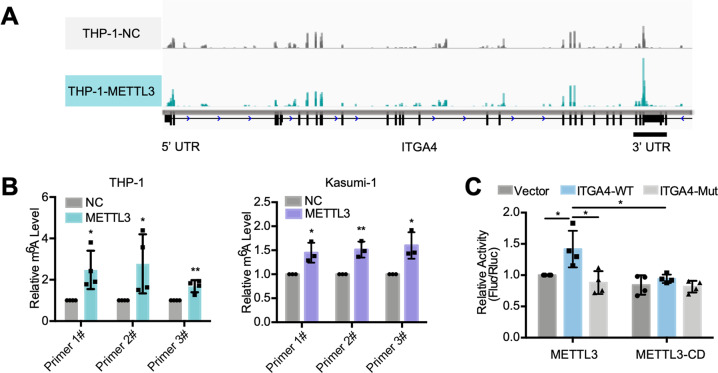
METTL3 regulates stability of ITGA4 mRNA. **A** The m^6^A abundances of ITGA4 mRNA transcripts in METTL3-overexpressing and control THP-1 cells as detected by m^6^A-seq (*n* = 3). Dotted boxes indicate regions for qPCR in (**B**). **B** Increasing m^6^A modification in specific regions of ITGA4 transcripts upon METTL3 overexpression as tested by gene-specific m^6^A-qPCR assays of THP-1 and Kasumi-1 cells. **C** Dual-luciferase reporter assays showing the effect of METTL3 and METTL3-CD on ITGA4 reporters with either wild-type or mutated m^6^A sites. *P < 0.05, **P < 0.01; *t* test. *n* ≥ 3, mean ± SD values are shown for (**B**) and (**C**).

Subsequently, the mechanisms by which METTL3 regulates the expression of ITGA4 were studied. The m^6^A-seq revealed that the vast majority of m^6^A peaks were distributed in the protein-coding region (CDS) and the 3’ untranslated region (3’ UTR) of mRNA transcripts in AML cells (Supplementary Fig. S10A–C). Notably, the m^6^A modification in the 3’ UTR of ITGA4 mRNA was significantly increased when METTL3 was overexpressed (Fig. 7A). Then, based on the sequence characteristics of ITGA4 mRNA given by m^6^A sequencing (Supplementary Fig. S10D), three pairs of primers for the 3’ UTR of ITGA4 mRNA were designed, and MeRIP-qPCR was carried out. The results showed that the amount of the 3’ UTR of ITGA4 mRNA that was pulled down by the m^6^A antibody was significantly increased when METTL3 was overexpressed (Fig. 7B), indicating that METTL3 methylated the 3’ UTR of ITGA4 mRNA. Finally, we found that the enhancement of ITGA4 mRNA expression by METTL3 was significantly weakened when the potential target sites in the 3’ UTR of ITGA4 mRNA were mutated (Fig. 7C and Supplementary Fig. S10E). Moreover, METTL3-CD had no similar regulatory effects. The results confirmed that METTL3 regulated the expression of ITGA4 mRNA by methylating specific 3’ UTR sites. Finally, we demonstrated that METTL3 overexpression significantly slowed the rate of ITGA4 mRNA degradation, whereas METTL3 knockdown or STM2457 pretreatment significantly accelerated the rate of ITGA4 mRNA degradation (Supplementary Fig. S10F). All the results confirmed that METTL3 increased the half-life of ITGA4 mRNA by m^6^A modification, then promoted the expression of ITGA4 on the cell surface and helped the cells access the BM niche.

## Discussion

Primary resistance to initial treatment and disease relapse are major limitations that have not been addressed for the treatment of AML [2]. Many efforts have been made to target primary and secondary AML chemoresistance, with limited success. METTL3-regulated m^6^A modification has been proven to be involved in the carcinogenesis of AML [12, 20], and a selective catalytic inhibitor of METTL3 has shown potential for AML treatment [22]. Notably, this study showed that the expression of METTL3 in AML patients was associated with adverse treatment outcomes and further confirmed that METTL3 regulated AML chemoresistance by mediating homing/engraftment and m^6^A methylation of ITGA4 mRNA, which might guide the clinical application of METTL3 inhibitors in the treatment of AML.

First, we found that METTL3-mediated m^6^A modification was an effective treatment target for AML chemoresistance. METTL3 has been proven to be involved in drug resistance in many cancers, but opposite roles have also been found in various cancer treatment models [38–41]. Specific to blood tumors, one report showed the regulation of FTO-dependent m^6^A demethylation on the targeted drug resistance of TKIs [16], but no evidence about the association between blood tumors’ METTL3 expression and BM-related chemoresistance has been reported. In addition, previous studies have confirmed that METTL3 is precisely and differentially regulated in different tumors and organs [42–47], elucidating the mechanism underlying the abnormal overexpression of METTL3 increase during relapse or primary resistance may be a key option for targeted intervention in AML. Regarding the relationships between METTL3 and AML, as early as 2017, Vu et al. found that depletion of METTL3 induced AML cell differentiation and apoptosis and delayed leukemia progression by mediating m^6^A methylation [12]. In the same year, Barbieri *et al*. proved that promoter-bound METTL3 introduced m^6^A methylation within the coding region of SP1 and SP2 transcripts to promote AML maintenance [20]. These reports led to increased interest in the use of METTL3 for the treatment of AML. Recently, Yankova et al. screened out a highly potent and selective catalytic inhibitor of METTL3 that exerted a significant therapeutic effect on AML in vitro and in vivo [22]. The discovery of STM2457 showed the therapeutic importance of METTL3 in AML and is an important step for the use of METTL3 as an AML treatment target. In our study, we found that METTL3 enhanced the IDA tolerance of AML cells in vitro and in vivo, and STM2457 exerted a notable inhibitory effect on the homing and engraftment of AML cells in mouse models. The treatment of chemoresistant AML patients with METTL3 inhibitors shows promise.

Second, we showed for the first time that METTL3 enhanced AML chemoresistance by regulating the homing and engraftment of AML cells, which provided a new strategy for targeting AML chemoresistance. AML cell homing to and engraftment in the BM are important for chemoresistance [48, 49]. Many molecules in AML cells and the BM microenvironment have been proven to mediate the homing and engraftment of cells [50, 51]. However, no report about the relationship between the m^6^A modification system and AML homing/engraftment has been found. A recent report on STM2457 found that, compared with the initial STM2457-treated mice, the mice after retransplantation of the AML cells that had been treated with STM2457 had a significant increase in lifespan and a marked decrease in the presence of AML cells in peripheral blood [22]. These results suggested that METTL3 might play an important role in the implantation of AML cells. Our results also showed that the enhancement of ITGA4 mRNA expression by METTL3 was significantly weakened when the potential m^6^A methylated target sites were mutated. In addition, METTL3 significantly affected the rate of ITGA4 mRNA degradation. All the results confirmed that METTL3 can increase the stability of ITGA4 mRNA transcript by m^6^A modification, then promoted the expression of ITGA4 on the cell surface and helped the cells access the BM niche. The study linked the m^6^A modification system with the BM microenvironment and showed that METTL3-mediated m^6^A modification affected the communication between cells and other cells or the microenvironment by regulating ITGA4. Why ITGA4 is selected as a key “signal detector” for METTL3-mediated homing of AML cells should be further studied.

Overall, our studies have identified a novel AML chemotherapy drug-resistant protein, METTL3, which can mediate AML cell homing and engraftment by m^6^A modification of ITGA4 mRNA. These findings provide insight into the mechanisms underlying AML chemoresistance and develop a novel chemoresistance regulatory system based on METTL3-mediated m^6^A modification. These findings will help further elucidate the precise regulatory mechanisms of AML chemoresistance and provide a new perspective for METTL3 as a novel treatment target of AML.

## Supplementary information

Supplementary Information

Supplementary Table 1

Supplementary Table 2

Supplementary Table 3

## Data Availability

The data that support the findings of this study are available from the corresponding author upon reasonable request. RNA-Seq and m^6^A-Seq raw data have been deposited in the National Center for Biotechnology Information Gene Expression Omnibus database (accession GSE212125).
