# Supplementary material for: METTL3 mediates chemoresistance by enhancing AML homing and engraftment via ITGA4
Source: Leukemia. 2022 Oct 20;36(11):2586–95. doi: 10.1038/s41375-022-01696-w (PMC9613467; doi:10.1038/s41375-022-01696-w)
Supplement: Supplementary file 1 — Supplementary Information [file 41375_2022_1696_MOESM1_ESM.pdf]

Supplementary Materials and Methods

Cell lines.

THP-1 cells were obtained from the China National Collection of Authenticated Cell Cultures and cultured in RPMI-1640 (Gibco, USA) with 10% fetal bovine serum (FBS) (Gibco); Kasumi-1 and HL-60 cells were obtained from the National Collection of Authenticated Cell Cultures and cultured in IMDM (Gibco) with 20% FBS; HL-60/ADR cells were obtained from the Institute of Hematology & Blood Diseases Hospital, Chinese Academy of Medical Science & Peking Union Medical College and kept in RPMI-1640 with 10% FBS and IMDM with 10% FBS; HEK293T cells were obtained from the China National Collection of Authenticated Cell Cultures and grown in DMEM (Gibco) with 10% FBS. These cell lines are not among the commonly misidentified cell lines and were authenticated by short tandem repeat (STR) analysis.

Lentivirus infection.

All the lentiviruses in this paper were packaged by GeneChem, Co., dCas9-VP46-Puro, GV419-sgRNA-Neo, GV358-3FLAG-EGFP-Puro and CV186-3FLAG-mCherry-Puro vectors were used. For lentivirus infection, METTL3 (GeneChem, China, SHCLNV: NM_019852, Gene ID:56639) concentrated virus was directly added to cells. The positively infected cells were selected with 1 μg/mL puromycin (Sigma-Aldrich, #P8833).

For generation of dCas9 stable cell lines, cells were transduced with dCas9-VP64-Puro lentivirus and then replaced with 2 mL of complete media containing puromycin. The stably screened cells were transfected with sgMETTL3-GV419-Neo (GeneChem, SHCLNV: NM_019852, Gene ID:56639) and its negative control lentivirus for

secondary transfection and then treated with G418 (Gibco, #10131) screening treatment. For the generation of single clones of THP-1-dCas9-sgMETTL3 and Kasumi-1-dCas9-sgMETTL3 cells, single cells were seeded in 96-well plates, and cells grown up from one single cell were selected.

RPPA.

Reverse Phase Protein Array (RPPA) was developed on THP-1 and THP-1/IDA cells by Mills Institute for Personalized Cancer Care, Fynn Biotechnologies Ltd.

Mouse studies and animal housing.

C57BL/6 or NSG mice were purchased from Beijing Vital River Laboratory Animal Technology Co., Ltd. All the animal experiments were carried out in accordance with the guidelines published by the Institutional Animal Care and Use Committee of Shandong University. All the animals were housed in an SPF animal house in the animal center of Qilu Hospital, Shandong University. MLL-AF9 secondary mouse leukemia cells were donated by the Institute of Hematology, Chinese Academy of Medical Sciences.

For the in vivo experiments shown in Fig. 3 and Supplementary Fig. S4, six- to eight-week-old male NSG mice were injected via the tail vein with three types of THP-1 cells (5×10^6 cells per mouse) that were transfected with viruses (THP-1-NC&-METTL3&-METTL3-CD cells). The treatment group was intraperitoneally treated with IDA (0.3 mg/kg per day for 7 days) beginning on day 42 after cell injection, and the control group was injected with PBS. An *in vivo* imaging system was used to quantitate disease burden by detecting up the GFP fluorescence signal. For the in vivo experiments shown

in Supplementary Fig. S5, six- to eight-week-old male C57BL/6 mice were injected by the tail vein with three different kinds of MLL-AF9 cells (5×10^6 cells per mouse) transfected with viruses. The treatment group was intraperitoneally treated with a daily dose of 50 mg/kg Ara-C for 5 days along with a daily dose of 1.5 mg/kg DOX during the first 3 days beginning on day 8 after cell injection. 1×10^5 BM cells and spleen cells were harvested and analyzed the percentage of GFP- or mCherry- positive cells was analyzed by flow cytometric analyses. Another batch of mice treated in the same way was used for survival analysis.

For the homing and engraftment experiments shown in Fig. 4 and Supplementary Fig. S7, three types of THP-1or MLL-AF9 cells that were transfected with viruses (-NC&-METTL3&-METTL3-CD cells) were injected into NSG or C57BL/6 mice via tail-vein injection (1×10^7 cells per mouse for homing analysis and 5×10^6 cells per mouse for engraftment). Sixteen hours after BM transplantation for homing experiments and 1 week after MLL-AF9 transplantation or 6 weeks after THP-1 transplantation for engraftment experiments, 1×10^5 BM cells and spleen cells were harvested and analyzed the percentage of GFP- or mCherry- positive cells was analyzed by flow cytometric analyses.

For the *in vivo* experiments shown in Fig. 5 and Supplementary Fig. S8, THP-1 cells and THP-1/IDA cells were treated with DMSO or STM2457 (MedChemExpress, Monmouth Junction, NJ, USA, #HY-134836, 20 μM), respectively, for 48 h before tail vein injection. Sixteen hours after transplantation for homing experiments and 6 weeks after transplantation for engraftment experiments. A total of 1×10^5 BM cells and spleen cells were obtained and stained with anti-hCD45-APC (BioLegend, San Diego, CA,

USA, #304012), followed by flow cytometry.

To establish mouse models of primary AML patients, we injected the cryopreserved leukemic blasts into NSG mice via tail-vein injection (2×10^6 cells per mouse, 2 mice per patient). The percentage of CD45+ cells in the bone marrow and spleen of euthanized animals was used to determine the extent of leukemia infiltration. The bone marrow cells were extracted from femurs and tibia by crushing the bone in medium, supplemented with 10% fetal bovine serum. To determine the response to chemotherapeutic drugs, the mice were intraperitoneally injected with either IDA (0.3 mg/kg per day for 7 days) or PBS by beginning on day 35. The IDA response was determined by flow cytometric analysis of CD45+ cells among the leukocytes isolated from the bone marrow and spleen of mice in the PBS- and IDA-treated groups. All the flow cytometric analyses were performed in Galias (Beckman Counter, Brea, Calif, USA), and all data were analyzed by using FlowJo 10.4.

EdU and CFA.

For EdU assays, an iClick™ EdU Andy Fluor™ 647 Flow Cytometry Assay Kit (ABP Biosciences, Rockville, MD, USA, #A008) was used, and the treated cells were tested by flow cytometry. Cells from different groups were seeded in 6-well plates at a concentration of 5×10^5 cells per well with PBS or IDA (0.005 μg/mL) for 24 h. For the TR-14035 rescue experiment, cells from different groups and treated with 10 μM TR-14035 (MedChemExpress, #HY-15770) or vehicle control DMSO combined with IDA (0.005 μg/mL) for 24 hours prior to EdU experiments. All data were analyzed by using Kaluza. For the colony-forming assay, treated cells were plated in methylcellulose medium (MethoCult™ H4230, Stem Cell Technologies, Canada, #04230) according to

the manufacturer's instructions. Colonies were evaluated and scored after 14–21 days of incubation.

Cell migration and adhesion experiments.

An 8 μm pore size Costar Transwell plate (Corning, NY, USA) was used to measure the migratory potential of AML cells. A total of 4×10^5 cells were washed and resuspended. For the TR-14035 rescue experiment, cells were suspended in 100 μL of RPMI-1640 medium containing 10 μM TR-14035 (MedChemExpress, #HY-15770) or vehicle control DMSO and seeded in the upper chamber. For the STM2457 rescue experiment in Fig. 5 and Supplementary Fig. S8, cells treated with 10 μM STM2457 (MedChemExpress, #HY-134836) or DMSO for 48h were suspended in 100 μL of RPMI-1640 medium and seeded in the upper chamber. Next, 500 μL of RPMI-1640 medium containing 10% FBS was added to the lower well with HUVECs. After incubation for 24 h, the migrated cells were measured by fluorescence microscopy and analyzed. For the detection of cell adhesion, a total of 1×10^5 AML cells were seeded in 6-well plates covered with HUVECs. After incubation for 4 h, the adhered cells were measured by fluorescence microscopy and analyzed.

RT-PCR.

Total RNA was extracted using TRIzol reagent (Thermo Fisher, #15596026) according to the manufacturer's protocol and was reverse-transcribed into cDNA using PrimeScript™ RT Master Mix (Takara, Japan, #RR036A). Quantitative real-time PCR was performed using TB Green Premix Ex Taq™ II (Takara, #RR820A) on a Light Cycler 480 II (Roche). GAPDH was used as endogenous control. The primer sequences were: GAPDH, AAG GTG AAG GTC GGA GTC AAC and GGG GTC ATT

GAT GGC AAC AAT A; METTL3, CGT ACT ACA GGA TGA TGG CTT TC and TTT CAT CTA CCC GTT CAT ACC C; ITGA4, CCA TCG TGA CTT GTG GGC A and GTA AAT CAG GGG GCA CTC CA; 18 S, GTA ACC CGT TGA ACC CCA TT and CCA TCC AAT CGG TAG TAG CG. The primer sequences for MeRIP qPCR were as follows: Primer 1, AAC AGA AGA GAC AGT TGG AGT T and TCA TGA GTA AAA GAA GTC CAA ACA A; Primer 2, CAT GCA AGG GGA AAA TCT CAG C and TAT CTT CAA GGG CTT ACT TTT CTT G; Primer 3, GGG CTG TGT TCC AAC AAC CA and ACA GAA AGA AAG ATG GTG AAA TCC A.

m^6A dot blot.

mRNA of AML cell sample was enriched by a Dynabeads mRNA Purification Kit (Invitrogen, #61006) from total RNA. mRNA samples in a volume of 2 μL were denatured by heating at 72°C for 5 min followed by immediate chilling on ice to prevent secondary structure remodeling of mRNA. Then, mRNA was loaded on the Amersham Hybond™-N+ membrane (GE Healthcare, Chicago, Illinois, USA, #RPN303B) and crosslinked to the membrane twice by UV in autocrosslink mode (1 200 microjoules [x100]; 25–50 sec) using UVP Crosslinker Analytik-jena. One of the membranes was blocked with 5% nonfat powered milk (Sangon Biotech) in TBST and detected with an m^6A-specific antibody (Synaptic Systems, Germany, #202003). The other membrane was stained with methylene blue as a loading control.

LC-MS/MS for determination of the m^6A/A ratio.

Double selected polyadenylated (poly[A]) mRNA was digested by nuclease phosphodiesterase I (0.1 U, Sigma), benzonase (10 U, Sigma) and alkaline phosphatase (1 U, Sigma) in 50 μL of buffer containing 50 mM Tris-HCl, 1 mM $MgCl_2$ (pH = 8.0)

and 100 μg/mL BSA following incubation at 37°C for 3 h. The samples were diluted to 50 μL, filtered (0.22 mm pore size, 4 mm diameter, Millipore) and then loaded onto a C18 reverse-phase column coupled online to an Agilent 6410 QQQ triple-quadrupole LC mass spectrometer in positive electrospray ionization mode. The nucleosides were quantified by using retention time and nucleoside to base ion mass transitions of 282.1–150.1 (m^6A), 268–136 (A). Quantification was performed in comparison with the standard curve obtained from pure nucleoside standards running with the same batch of samples. The m^6A level was calculated as the ratio of m^6A to A.[1]

RNA-seq and m^6A-seq.

After 7 days of screening stably transfected cell with puromycin (1 μg/mL), THP-1-NC and THP-1-METTL3 cells were harvested separately for the subsequent downstream analysis of METTL3. Total RNA was extracted using TRIzol reagent (Thermo Fisher) following the manufacturer's instructions, and the RIN number was determined to evaluate the RNA integrity by an Agilent Bioanalyzer 2100 (Agilent Technologies). Qualified total RNA was further purified by an RNAClean XP Kit (Beckman Coulter) and RNase-Free DNase Set (QIAGEN).

For RNA-seq, a VAHTS Universal V6 RNA-seq Library Prep Kit for Illumina® (Vazyme, #NR604-01) was used to establish the library. Each group was sequenced with an Illumina HiSeq 2000, and the single-end sequencing length was 200-300 bp. Sequence reads were compared with version 38 of the human genome (GRCH38) using a standard Illumina sequence analysis pipeline.

For m^6A-seq, total RNA was fragmented into 100-200 nt RNA fragments using $ZnCl_2$

and incubated with anti-N6-Methyladenosine (m^6A) antibody (Sigma Aldrich, #SAB5600251) Then, the products above were mixed with protein A/G magnetic beads (Thermo Fisher, #88803) at 4 ℃. A NEBNext Ultra RNA library prep kit (NEB, #E7775) was used to establish the library for the purified mRNA fragments. Each group was sequenced with an Illumina NovaSeq 6000 sequencer, and the single-end sequencing length was 150 bp. Reads were compared with human genome version GRCH38 through TopHat. ExomePeak ($P < 0.01$) was used to identify differential m^6A-modified peaks between IP and input samples.

RNA stability.

Cells were treated with actinomycin D (Sigma-Aldrich, #A9415) at a final concentration of 5 mg/mL for 0, 1, or 2 h/0, 1, 2, 3 or 4 h and collected. Cells were harvested at the indicated time points, and total RNA was extracted and tested by qRT-PCR. Relative mRNA levels were normalized to the starting point of treatment.

Western blot.

Cells were lysed in M-PER™ Mammalian Protein Extraction Reagent (Thermo Fisher, #78501) with protease inhibitor cocktail (Roche). The membranes were incubated sequentially with the diluted (1:1 000 or 1:500) primary and secondary antibodies. The following antibodies were used to perform: METTL3/MT-A70 antibody (Bethyl, Waltham, MA, USA, #A301-568A), Integrin Alpha 4 (Cell Signaling Technology, #8440), beta Tubulin Antibody (Thermo Fisher, # 480011).

Dual-luciferase reporter assays.

The ITGA4–3'UTR containing the WT m^6A motifs and mutant motifs (m^6A was

replaced by T, see Supplementary Fig. S10E) was synthesized by Integrated DNA Technologies and subcloned into the HindIII-SacI site of the pMIR-REPORT™ firefly luciferase reporter vector (Ambion, #GN1399). For dual-luciferase reporter assays, 400 ng WT or mutant firefly luciferase reporters (e.g., ITGA4-WT, ITGA4-mut), 500 ng METTL3 expression vectors (e.g., METTL3-WT-pcDNA3, METTL3-CD-pcDNA3, or pcDNA3), and 100 ng pRL-TK Renilla luciferase reporter vector (Promega, USA) were cotransfected into HEK293T cells in 24-well plates using Lipofectamine 2000 (Invitrogen). The luciferase activities were assessed 48 h posttransfection by the Dual-Luciferase Reporter Assay System (Promega, #E2920) and analyzed.

Statistics.

Sample size was chosen $n \geqslant 3$. Data were analyzed and presented as the mean ± SD. Unpaired t test and correlativity test were used to analyze the two groups as indicated; two-way ANOVA was used to compare the inter- and intra- differences of two factors in two groups as indicated; $P < 0.05$ was considered significant. Statistical analyses were performed using GraphPad Prism 9.0 or the R statistical environment. For Fig. 3C and Supplementary Fig. S5G, Kaplan-Meier survival curves were generated using GraphPad Prism 6, and the *P* values were calculated using the log rank test.

Supplementary Figures and Figure Legends

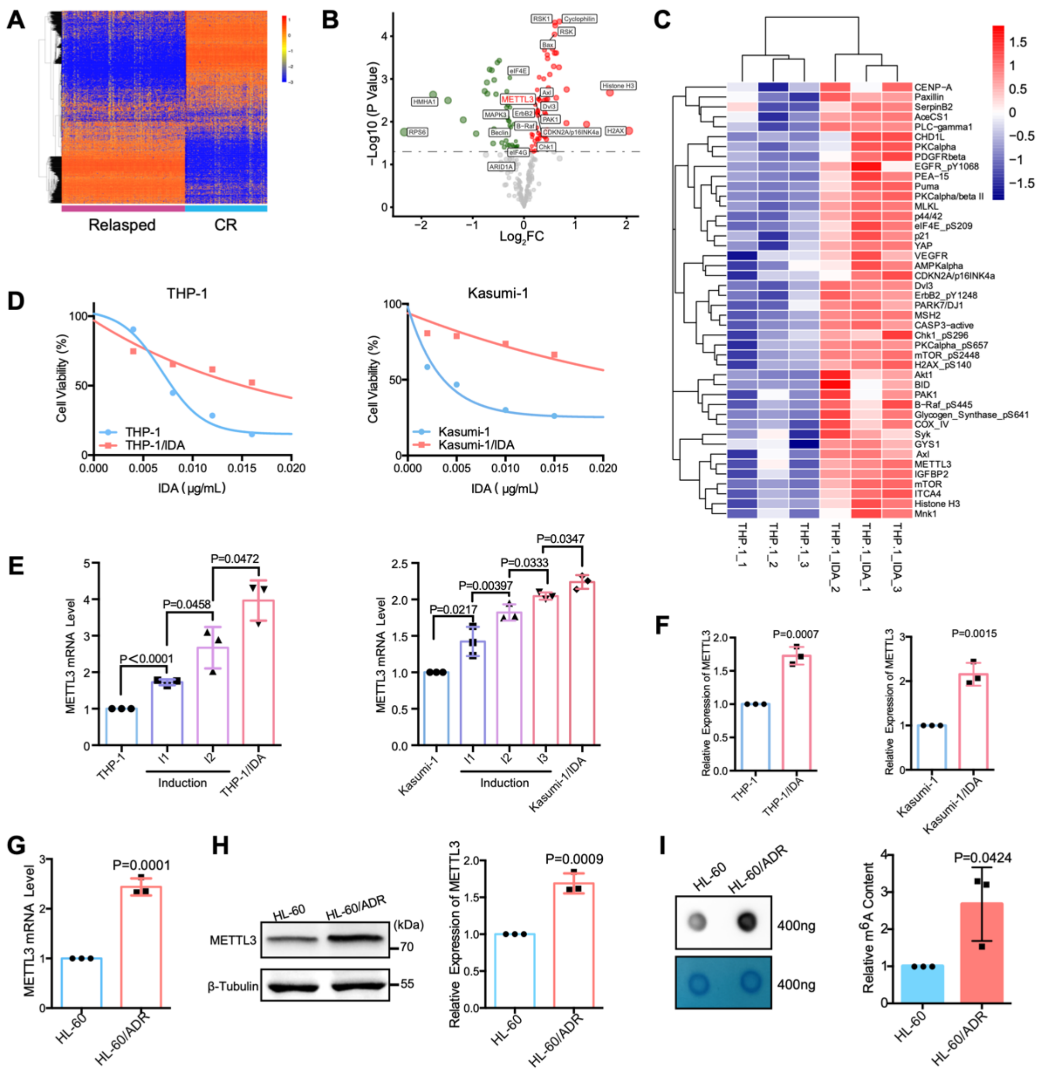

Fig. S1 Screening process of METTL3 and its expression in chemotherapy-resistant cell lines. A Heatmaps showing the upregulated and downregulated genes from GES165430 (n=268). **B** Volcano plot depicting proteins enriched in THP-1/IDA cells (red) and THP-1 cells (green) analyzed by RPPA ($P < 0.05$). **C** Heatmaps showing perturbed protein expression with significant increase in chemotherapy resistant cells by RPPA (LogFC > 0.25). **D** CCK-8 assays of THP-1&THP-1/IDA cells and Kasumi-1&Kasumi-1/IDA cells treated with IDA for 48 h (n=3). **E** qRT-PCR analysis showing METTL3 expression at different induction periods in the chemotherapy-resistant cell lines THP-1 and Kasumi-1. n=3, $P < 0.05$ was considered significant, t test. **F** Statistical

results of Western blot analysis for the expression of METTL3 in THP-1&THP-1/IDA cells and Kasumi-1&Kasumi-1/IDA cells (*n*=3). **G** qRT-PCR analysis of METTL3 expression in HL-60/ADR cells (*n*=3). **H** Western blot analysis for the expression of METTL3 in HL-60/ADR cells and the statistical results (*n*=3). **I** HL-60&HL-60/ADR cells were subjected to mRNA extraction followed by m^6A dot blot assays to detect global m^6A changes and summarized. *n*=3, MB, methylene blue staining (as loading control). Mean ± SD values are shown for (**E**)-(**I**). $P < 0.05$ was considered significant; t test.

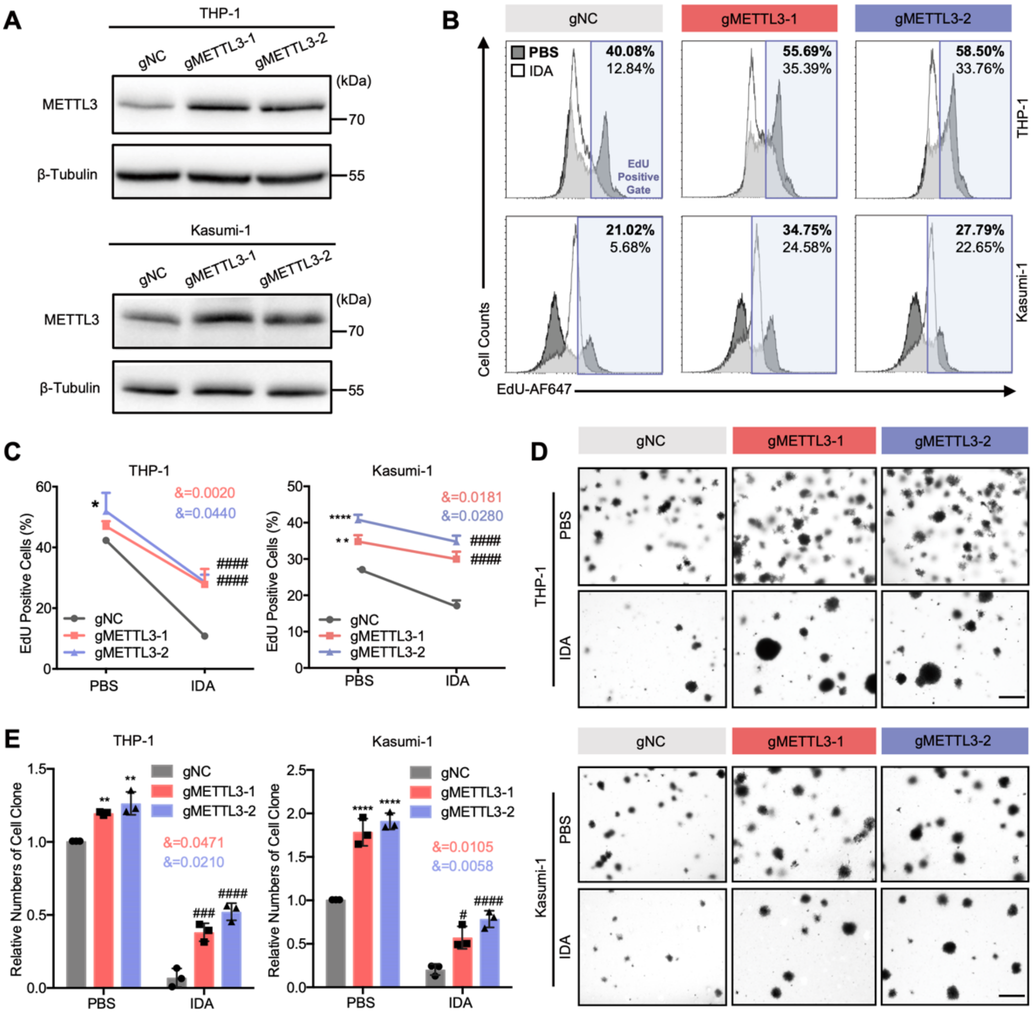

Fig. S2 The overexpression of endogenous METTL3 confers chemoresistance to AML cells. A-C The overexpression efficiency was confirmed by Western blot and shown in (**A**). EdU incorporation assay showing the percentage of AML cells (n=3) entering the proliferation cycle (EdU positive cells) with or without IDA pressure (**B**) and are summarized (**C**). **D-E** Colony-forming assay (**D**) showing images of AML cells with or without IDA pressure. Bar, 500 μm. Statistical analysis is shown in (**E**). Mean ± SD values are shown for (**C**) and (**E**). *, $P < 0.05$, **, $P < 0.01$, ****, $P < 0.0001$, vs. the NC group with PBS treatment; $^{\#}$, $P < 0.05$, $^{\#\#\#}$, $P < 0.001$, $^{\#\#\#\#}$, $P < 0.0001$, vs. the NC group with IDA treatment; &, significant interaction effect; two-way ANOVA.

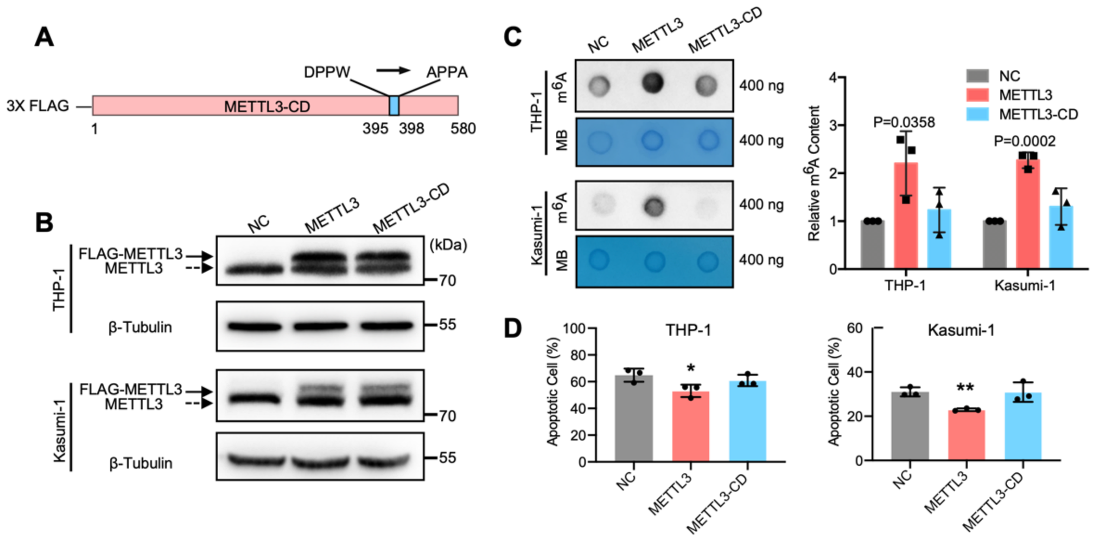

Fig. S3 The overexpression of exogenous METTL3/METTL3-CD and corresponding mRNA m^{6}A modification confirmation. A Construction of METTL3-CD vectors. **B-C** Western blot (**B**) confirmation and corresponding changes in the mRNA m^{6}A expression levels (**C**) were measured by m^{6}A dot blot assays (left) and summarized (right). Data are mean ± SD values. $P < 0.05$ was considered significant, t test. **D** Percentage of apoptotic cells in a THP-1 and Kasumi-1 cells from three replicate experiments. n=3, Mean ± SD values are shown for (**C**) and (**D**). *, $P < 0.05$, **, $P < 0.01$, t test.

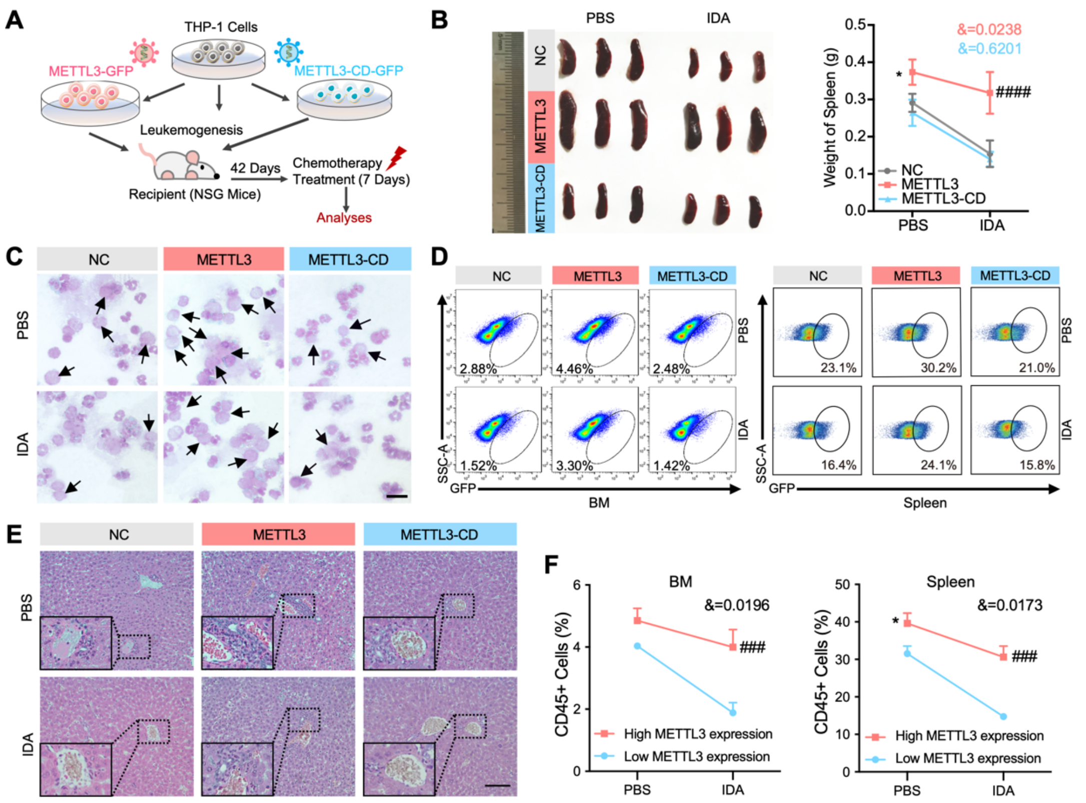

Fig. S4 METTL3 reduces the sensitivity of AML cells to chemotherapeutics in xenograft model. **A** Scheme of the design and procedures of the chemotherapy treatment sensitivity test with AML mice using NSG mice. **B** Spleen image and weight index from xenograft recipient mice with or without IDA treatment at the end point. *, $P < 0.05$, vs. the NC group with PBS treatment; $^{\#\#\#\#}$, $P < 0.0001$, vs. the NC group with IDA treatment; &, significant interaction effect; two-way ANOVA. **C**-**D** Wright-Giemsa staining of BM of the xenograft recipient mice at the end point is shown in (**C**); and the engraftment of THP-1 cells in BM and spleen of the NSG mice is shown in (**D**). Bar, 50 µm. **E** H&E staining of the liver of the xenograft recipient mice at the end point. Bar, 200 µm. **F** CD45+ cells infiltration in the high METTL3 expression group (n=3) and the low METTL3 expression group (n=3) of patient-derived xenografts (PDX models) with or without IDA treatment. Mean ± SD values are shown for (**B**) and (**F**).

*, $P < 0.05$, vs. the low METTL3 expression group with PBS treatment; ###, $P < 0.001$, vs. the low METTL3 expression group with IDA treatment; &, significant interaction effect; two-way ANOVA.

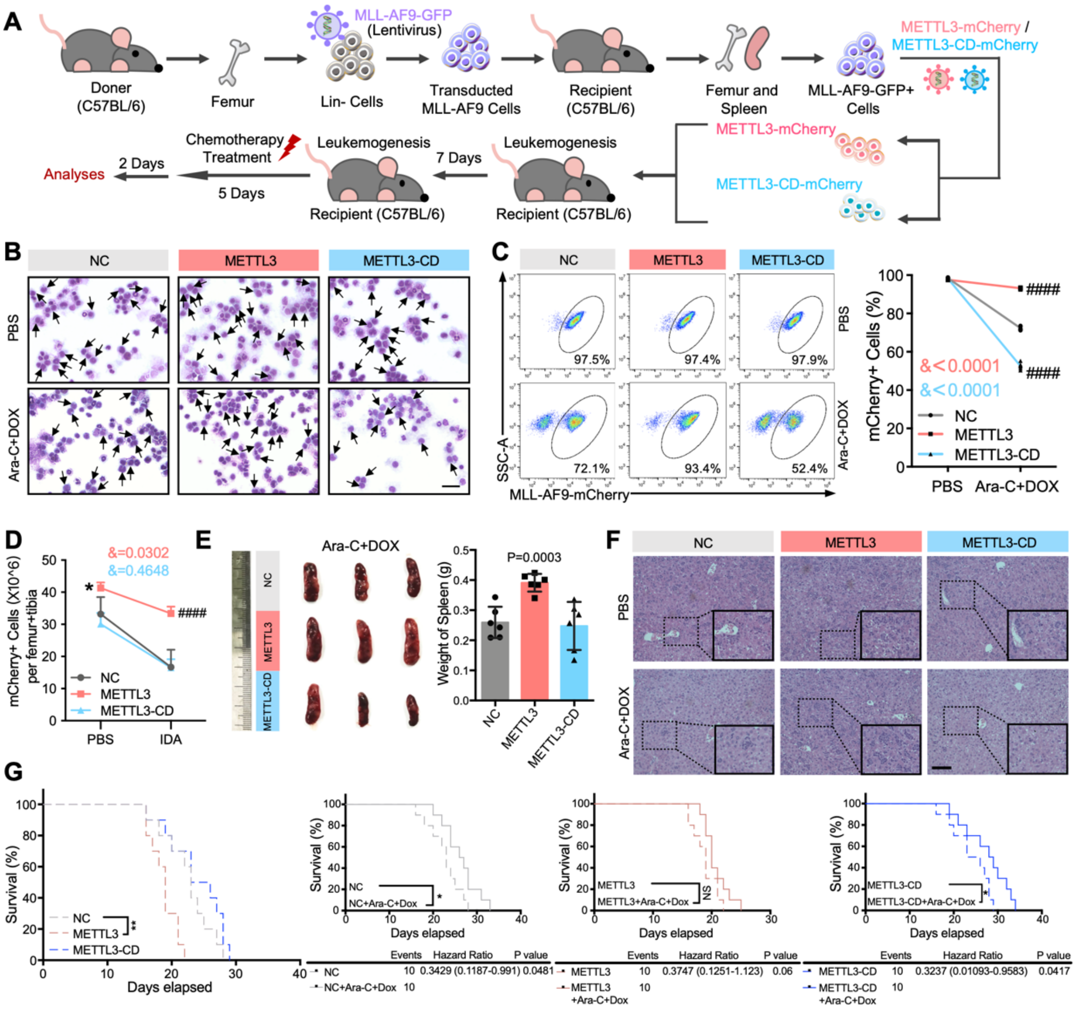

Fig. S5 METTL3-meditated m^{6}A reduces the sensitivity to chemotherapeutics of MLL-AF9+ AML mice. **A** Schematic outline of the experimental strategy of primary and secondary BMT using MLL-AF9 leukemic cells. **B** Wright-Giemsa staining of BM of the BMT recipient mice with or without Ara-C and DOX treatment on the 14th day. **C** and engraftment of MLL-AF9 cells in BM of the BMT recipient mice with or without chemotherapy treatment is shown and summarized. Bar, 50 μm. $^{####}$, $P < 0.0001$, vs. the NC group with PBS treatment; &, significant interaction effect; two-way ANOVA. **D** Absolute counts of MLL-AF9 cell infiltration in the BM of BMT recipient mice with or without chemotherapy. *, $P < 0.05$, vs. the NC group with PBS treatment; $^{####}$, $P < 0.0001$, vs. the NC group with IDA treatment; &, significant interaction effect; two-

way ANOVA. **E** Spleen image and weight index of BMT recipient mice treated with Ara-C and DOX at the end point. $P < 0.05$ was considered significant, t test. **F** H&E staining of the livers of the BMT recipient mice at the end point. Bar, 200 μm. **G** Kaplan-Meier survival curves showing the effects of forced METTL3 expression on the progression of MLL-AF9 cells in BMT recipient mice. *, $P < 0.05$, **, $P < 0.01$; NS, nonsignificant; t test. $n \geqslant 5$, mean ± SD values are shown for (**C**) and (**D**)-(**E**).

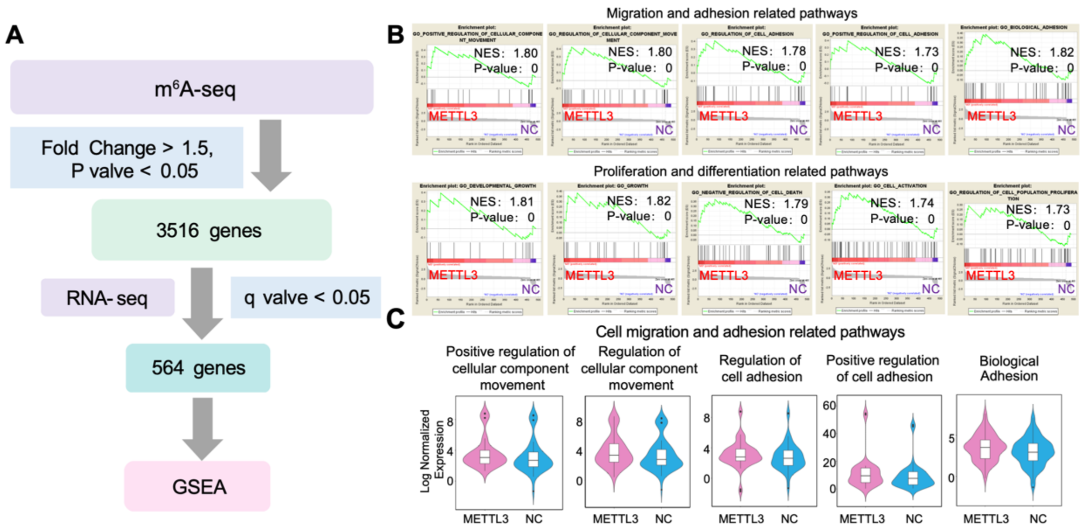

Fig. S6 METTL3 downstream analysis and GSEA A Schematic of METTL3 downstream analysis. **B** GSEA in THP-1 cells. Representative gene sets that were significantly enriched in the METTL3 overexpression samples (left panel) or the control samples (right panel) are shown. METTL3, METTL3 overexpression; NC, control; NES, normalized enrichment score; FDR, false discovery rate. **C** Violin plots showing the relative abundance of genes involved in the indicated migration/adhesion-related pathways in METTL3-overexpressing or control THP-1 cells.

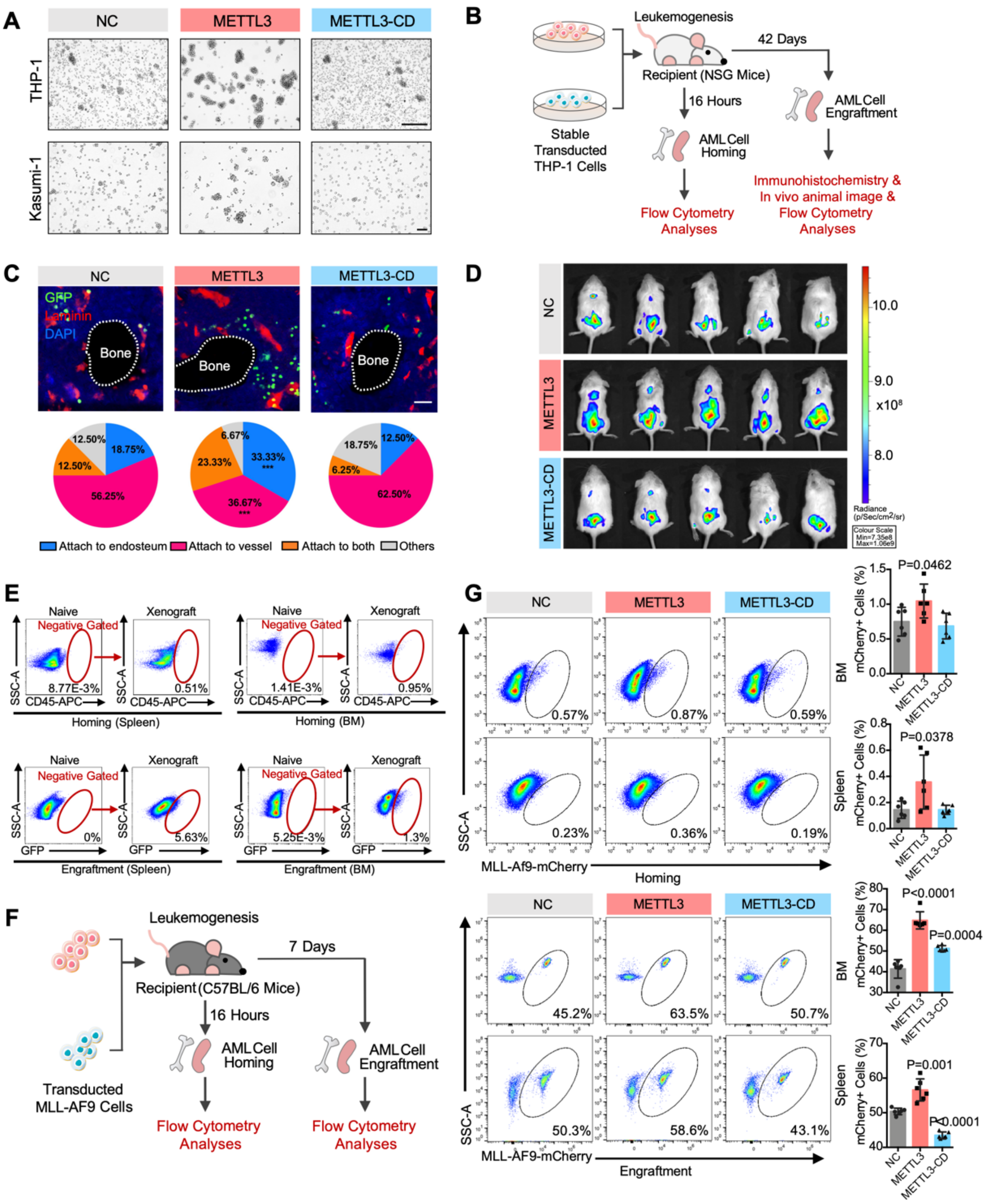

Fig. S7 AML homing and engraftment in xenograft/homograft recipient mice. A Images showing that METTL3-overexpressing AML cells have clustered growth characteristics. **B** Scheme of the design and procedures of the homing/engraftment test in AML xenograft recipient mice. **C** Representative images of localizations of THP-1 cells (GFP) in the vasculature (laminin, red) of the recipients (top); percentages of THP-1 cells attached to the endosteal niche, vascular niche, or both in (bottom). **D** *In vivo* animal imaging was carried out on the xenograft mice 42 days after tail vein injection.

E The flow cytometry gate principle for homing and engraftment of THP-1 cells in the spleen and bone marrow (BM) of NSG mice. **F** Scheme of the design and procedures of the homing/engraftment test in AML homograft recipient mice. **G** Flow cytometry was carried out for the homograft recipient mouse BM and spleen 16 h/7days after tail vein injection. The statistical results are shown on the right. $P < 0.05$ was considered significant vs. the NC group, t test. $n \geqslant 5$, mean ± SD values are shown for (**G**).

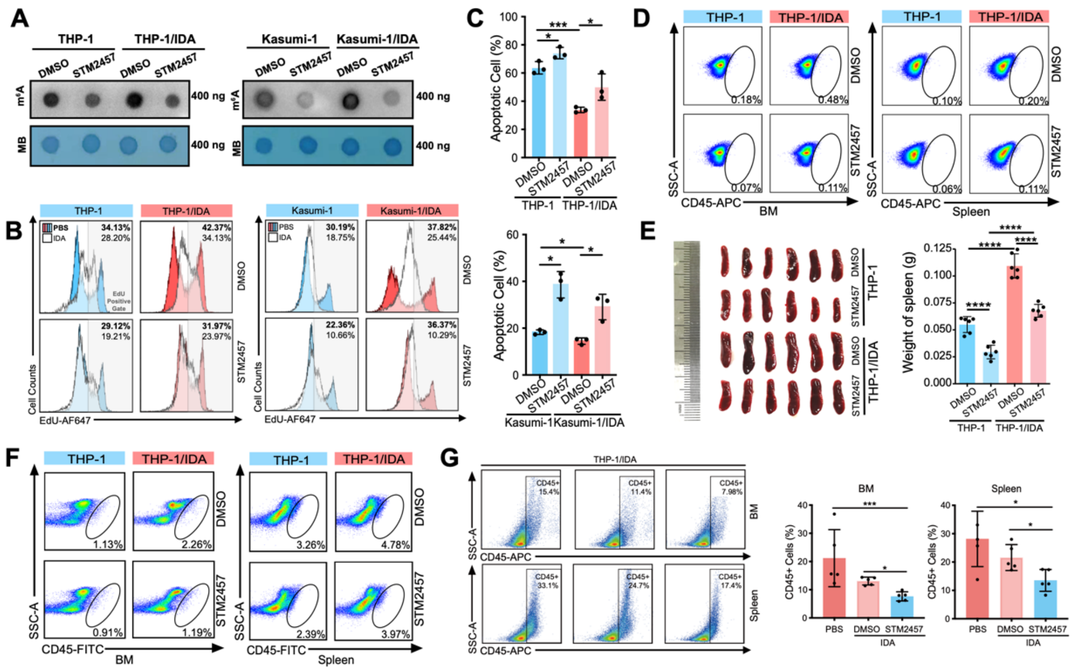

Fig. S8 A METTL3 inhibitor reverses AML chemoresistance and homing/engraftment capacity. A THP-1&THP-1/IDA and Kasumi-1&Kasumi-1/IDA cells with or without STM2457 treatment for 48 h were subjected to mRNA extraction followed by m^{6}A dot blot assays to detect global m^{6}A changes. MB, methylene blue staining (as loading control). **B** EdU incorporation assay showing the improvement of AML proliferation can be reversed by the pretreatment of STM2457 with or without IDA pressure. Percentages after PBS treatment are shown in bold, and percentages after IDA treatment are shown in regular. **C** The percentage of apoptotic cells showed that METTL3 inhibitor treatment promoted the apoptosis of AML cells (n=3). Data are mean ± SD values. *, P<0.05, ***, P<0.001, t test. **D** The homing of THP-1&THP-1/IDA cells with or without STM2457 pretreatment in the BM and spleen measured by flow cytometry 16 h after tail vein injection. **E** Spleen image (left) and weight index (right) from xenograft recipient mice (n=6) 42 days after tail vein injection. Data are mean ± SD values. ****, P < 0.0001; t test. **F** The engraftment of THP-1&THP-1/IDA cells with or without STM2457 pretreatment in the BM and spleen

measured by flow cytometry 42 days after tail vein injection. **G** STM2457 rescued the sensitivity of THP-1/IDA cells (*n*=5) *in vivo*. Statistical analysis is shown in right. Data are mean ± SD values. *, $P < 0.05$, ***, $P < 0.001$; t test.

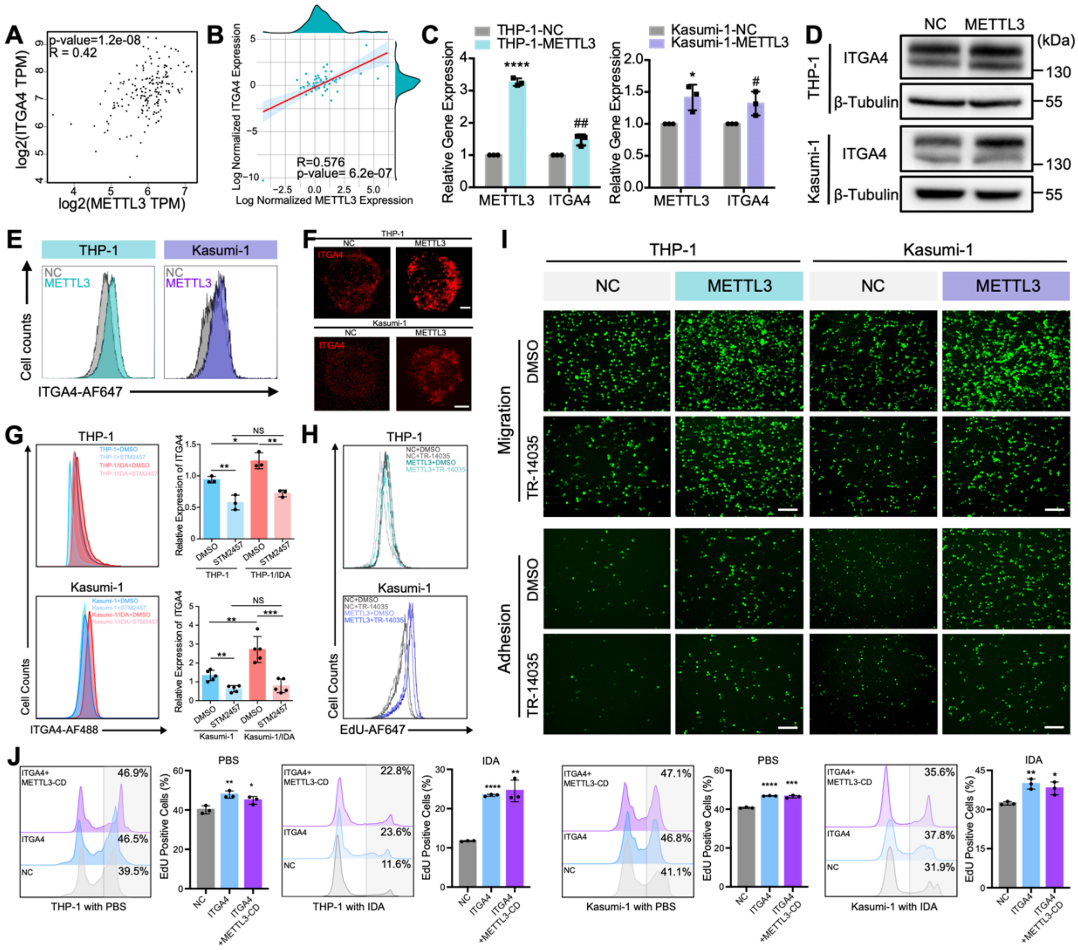

Fig. S9 METTL3 mediates AML chemoresistance by regulating ITGA4. A-B The Pearson correlation of ITGA4 with METTL3 mRNA expression across the AML samples in TCGA (**A**) and clinical data (**B**). $P < 0.05$ was considered significant. **C-F** Changes in ITGA4 expression are shown by qPCR analysis (**C**), Western blot analysis (**D**), flow cytometry (**E**), and immunofluorescence (**F**). Bar, 5 µm. *, $P < 0.05$, ****, $P < 0.0001$, METTL3 expression vs. the NC group; $^{\#}$, $P < 0.05$, $^{\#\#}$, $P < 0.01$, ITGA4 expression vs. the NC group, t test. **G** Changes in ITGA4 expression with or without STM2457 treated are shown by flow cytometry. Statistical analysis is shown on right. *, $P < 0.05$, **, $P < 0.01$; ***, $P < 0.001$, NS, nonsignificant; t test. **H** EdU incorporation assay showing that the improvement in the percentage of METTL3-overexpressing AML cells entering the proliferation cycle with IDA pressure can be partly reversed by TR-14035. **I** Images showing that the improvement of migration and

adhesion in METTL3-overexpressing AML cells can be partly reversed by TR-14035.

J EdU incorporation assay showing ITGA4 overexpression promoted AML chemoresistance. *, $P < 0.05$, **, $P < 0.01$, ***, $P < 0.001$, ****, $P < 0.0001$; t test. $n \geqslant 3$, mean ± SD values are shown for (**C**), (**G**) and (**J**).

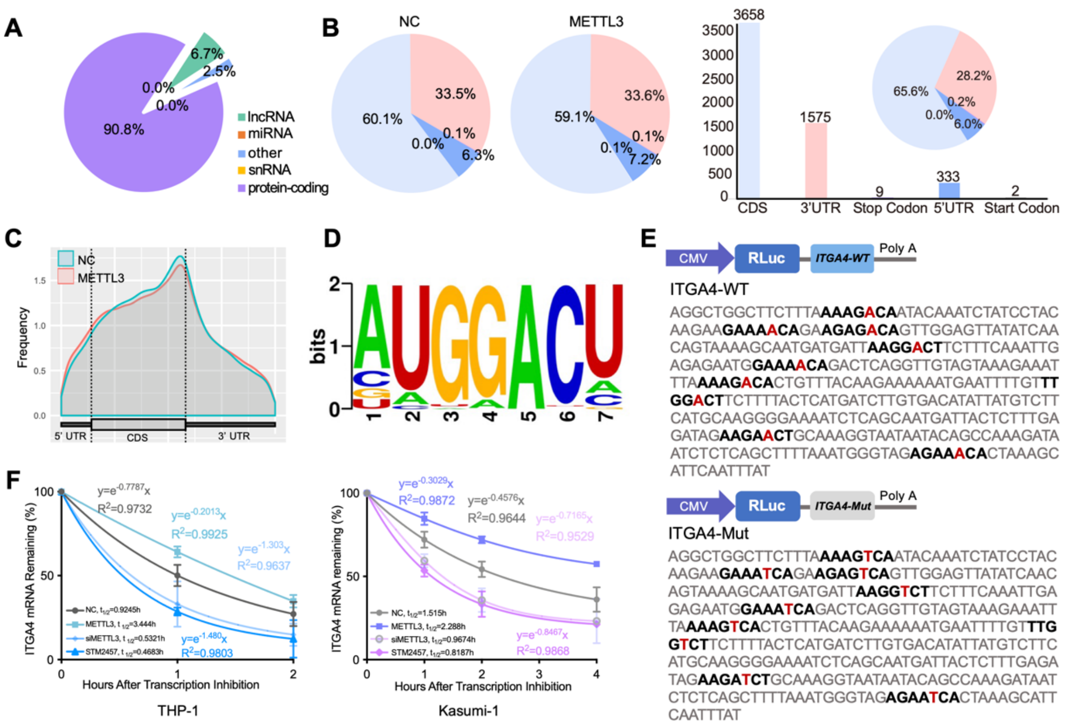

Fig. S10 METTL3 regulates stability of ITGA4 mRNA. A Pie charts showing the distribution of m^{6}A-seq reads in RNA classes. **B** The distribution of total m^{6}A peaks in the indicated regions of mRNA transcripts in the control and METTL3-overexpressing cells. **C** The proportion of m^{6}A peak distribution in the 5' UTR, start codon, CDS, stop codon or 3' UTR across the entire set of mRNA transcripts. **D** The predominant consensus motif DRACH ([G/A/U] [G/A] m^{6}AC [U/A/C]) detected by HOMER in m^{6}A-seq. **E** Construction of luciferase reporter vectors. The positions of the ITGA4 sequences used relative to their transcription start sites are shown. Putative m^{6}A consensus sequences are in bold, while the mutation sites are in red. **F** The mRNA half-life ($t_{1/2}$) of ITGA4 transcripts in THP-1 and Kasumi-1 cells with STM2457 inhibition or METTL3 knockdown/overexpression THP-1 and Kasumi-1 cells.

References

1. Su D, Chan CT, Gu C, Lim KS, Chionh YH, McBee ME, *et al.* Quantitative analysis of ribonucleoside modifications in tRNA by HPLC-coupled mass spectrometry. *Nat Protoc* 2014 Apr; **9**(4)**:** 828-841.
